# Treatment of Dysentery

**Published:** 1892-07

**Authors:** 


TREATMENT OF DYSENTERY.—Drs. Lardier and Pernet (Semaine Medicale) recommend the following treatment of dysentery—one which they have employed with the greatest success in the recent epidemic at Rambersvillers, Voges:

It is based on intestinal antisepsis, either by means of salol, or better still, iodoform. The former is prescribed in the following formula:

Salol, gr. xlv, (3.0 grammes.)
Tulu tincture, fl. dr. iiss, (10.0 grammes.)
Quince Syrup, fl. dr. vi, (30.0 grammes.)
Opium extract, gr. iss, (0.1 gramme.)
Mucilage acacia, oz. iv, (150.0 grammes.)

Tablespoonful hourly.

This medication is reported to act in a very favorable manner on the morbid process. However, the medicament which was found to have truly remarkable effects in dysentery, is iodoform, in the daily dose of 30 to 40 centigrammes, (4 1-2–6 grains.) The following formula is given:

Iodoform, gr. xv, (1.0 gramme.)
Powdered opium, gr. ix, (0.6 gramme.)

For 20 wafers—5 or 6 during the day, at equal intervals.

These wafers soon produced marked relief in the author's cases.

The incessant and extremely violent tenesmal pains were calmed by boric acid enemas; but the best means of combating them was found to be the use of suppositories of the following composition:

Cocaine hydrochlorate, gr. vi, (0.4 gramme.)
Powdered opium, gr. ix, (0.6 gramme.)
Cacao butter, dr. ii, (8.0 grammes.)
Divide into 4 suppositories, one morning and evening.

Under the influence of the cocaine, the griping disappeared as if by charm, the diarrhœa ceased, and the patients obtained a refreshing sleep of several hours' duration.

Besides the use of the medicaments mentioned, it is advised not to neglect washing out the intestines several times daily, with some antiseptic fluid—such as a concentrated solution of boric acid, or a 1:5000 solution of corrosive sublimate.—*The Med. Age.*

